# Efficacy of FOXP3+Treg cells combined with platelet in predicting recurrence of cervical cancer: a retrospective study

**DOI:** 10.1186/s12905-026-04274-9

**Published:** 2026-02-09

**Authors:** Shaoju Min, Luhong Xie, Yuting Wu, Li Bo, Yameng Liu, Yurong Zhu, Yujie Tan

**Affiliations:** 1 Centre for Clinical Laboratories, Affiliated Hospital of Guizhou Medical University, 28 Guiyi Street, Yunyan District, Guiyang, 550004 China; 2 School of Clinical Laboratory Science, Guizhou Medical University, Guiyang, China; 3 Clinical Laboratory, Guizhou Hospital, The First Affiliated Hospital of Sun Yat-sen University, Guiyang, China; 4 Department of Pathology, Affiliated Hospital of Guizhou Medical University, Guiyang, China; 5 Department of Pathology, Guizhou Medical University, Guiyang, China

**Keywords:** Cervical cancer, Tumor stroma tumor-infiltrating immune cells, Tumor center tumor-infiltrating immune cells, FOXP3 + Treg cells, Platelet, CD4^+^ t-cell exhaustion

## Abstract

**Background:**

Research on the impact of tumor-infiltrating immune cells(TIICs) combined with systemic inflammatory response (SIR) factors on cervical lesions and the prognosis of squamous cell cervical cancer (SCC) is limited. Therefore, this study aimed to evaluate the predictive and prognostic significance of TIICs and SIR factors in cervical epithelial lesions, specifically non-cervical epithelial lesions (NC), high-grade squamous intraepithelial lesions (HSIL), and SCC.

**Methods:**

This retrospective study analyzed 163 patients in three cohorts: NC (*n* = 59), HSIL (*n* = 52), and SCC (*n* = 52). Tumor-infiltrating immune cells (TIICs) in the tumor/lesion center and adjacent stroma were assessed via immunohistochemistry and multiplex immunofluorescence, while systemic inflammatory response (SIR) factors were derived from preoperative blood counts. The primary outcome was relapse-free survival (RFS) in the SCC cohort, analyzed using Cox proportional hazards regression.

**Results:**

TIICs were significantly elevated in the HSIL group compared with those in the NC group, accompanied by reduced platelet counts (PLT). The tumor stroma (TS) exhibited a greater degree of TIICs than the tumor/lesion center (TC) in both the HSIL and SCC groups. The presence of CD163+, CD11b+, and FOXP3 + TIICs, along with PLT levels, emerged as key indicators associated with the advanced histological stage. Compared to tTIICs, sTIICs demonstrated superior diagnostic performance in differentiating between HSIL and SCC groups. Lower levels of PLT (hazard ratio [HR] = 5.047, 95% confidence interval [CI]:1.373–18.540, *P* = 0.015), higher CD4 + T cells (HR = 0.211, 95%CI:0.062–0.722, *P* = 0.008), and FOXP3 + regulatory T cells (Tregs) (HR = 0.245, 95%CI:0.073–0.820, *P* = 0.010) were identified as poor prognostic indicators for recurrence-free survival (RFS) in SCC. A combination of FOXP3 + Tregs and PLT provided a more robust prediction of SCC recurrence. An increase in exhausted CD4 + T cells likely explains the observation that higher CD4 + T-cell infiltration correlated with lower RFS in SCC.

**Conclusion:**

The spatial distribution of TIICs, particularly the density in the tumor stroma, increases across the histological spectrum of cervical lesion severity. A signature combining FOXP3 + Treg cells and preoperative platelet counts provides a robust model for predicting SCC recurrence. Furthermore, the accumulation of exhausted CD4 + T cells appears to be a hallmark of disease advancement and poor prognosis, offering potential targets for personalized immunotherapy.

**Supplementary Information:**

The online version contains supplementary material available at 10.1186/s12905-026-04274-9.

## Background

 High-risk human papillomavirus (HR-HPV) infection is responsible for more than 99.7% of cervical cancers and poses a significant health threat to women [1]. Although cervical cytology screening and prophylactic vaccination offer the potential for elimination, the survival rate for patients with cervical cancer remains low. This is attributable not only to limitations in treatment advancements but also, critically, to delays in diagnosis which often preclude curative intervention [2, 3]. The prolonged carcinogenesis and pathological evolution of squamous cell cervical cancer (SCC) are influenced by the host’s baseline immunosurveillance and the gradual loss of immune control under persistent HR-HPV infection.

Some tumor-infiltrating immune cells (TIICs) subtypes are associated with tumor cell attack and immune suppression in SCC [4–6]. A significantly lower density of peritumoral CD20 + B cells has been observed in patients with relapsed SCC [7]. Simultaneously, virus-infected cells survive using immune escape strategies, including the induction and recruitment of immunosuppressive lymphocytes such as myeloid-derived suppressor cells (MDSCs), regulatory T cells (Tregs), and tumor-associated macrophages. However, the association between high FOXP3 + Treg infiltration and poorer disease outcomes in cervical cancer shows considerable between-study heterogeneity. This variability is largely attributed to the lack of standardized protocols for spatial evaluation (e.g., distinguishing between stroma and tumor center), the use of diverse phenotypic markers across different cohorts, and the scarcity of integrated analyses that combine both local immune cell infiltration and systemic inflammatory status, especially in regions with high disease burden [8, 9]. Therefore, further research is needed to clarify TIICs infiltration patterns and key prognostic factors in SCC.

High-grade squamous intraepithelial lesions (HSIL), including cervical intraepithelial neoplasia II and III, are referred to as immediate precursors of SCC and usually require aggressive treatment [10, 11]. Current treatment methods for HSIL are primarily surgical. While more thorough resection techniques can reduce treatment failure, they also increase the risk of premature delivery [12, 13]. Although tumor immunotherapy has shown promising results in various cancers, limited research exists on its potential to mitigate the long-term risks associated with surgical treatment for HSIL. Thus, understanding the infiltration pattern of TIICs may provide valuable insights into the immunological characteristics that distinguish SCC from HSIL [14].

Exhausted tumor-infiltrating T cells, marked by the upregulation of programmed cell death protein 1 (PD-1), contribute to tumor progression [15]. While extensive research has focused on exhausted CD8 + T cells, the role of exhausted CD4 + T cells in cancer is gaining attention. In multiple myeloma, a high frequency of PD-1 + CD4 + T cells with reduced proliferative potential in bone marrow samples has been associated with primary drug resistance [16]. In ovarian cancer, blocking the PD-1 pathway can restore the helper function of tumor-infiltrating PD-1^hi^CD39^+^ CD4 + T cells [17]. In peripheral blood, miR-568 inhibits the activation and function of CD4 + T cells by targeting the nuclear factor of activated T cells 5 (NFAT5), a key transcription factor linked to T-cell exhaustion differentially expressed in chronic infections versus malignant tumors [18]. However, it remains unclear whether CD4 + T-cell exhaustion occurs during the carcinogenesis of cervical cancer.

In addition to TIICs, cancer-related systemic inflammatory response (SIR) factors play a significant role in carcinogenesis. Peripheral circulation is a major source of immune cells migration, which contributes to the formation of TIICs. These markers are recognized prognostic indicators for chronic inflammation-related diseases and various solid malignant tumors [19]. However, research on the impact of SIR factors on cervical lesions and the prognosis of SCC is limited.

We designed a study spanning the histologic spectrum from benign tissue to invasive cancer. First, we performed a comparative analysis to characterize the changes in tumor-infiltrating immune cells (in both the tumor/lesion center [TC] and tumor stroma [TS]) and systemic inflammatory response (SIR) factors across non-cervical lesions (NC), HSIL, and SCC groups. Then, leveraging this baseline, our primary aim was to identify among these factors those that independently predict relapse-free survival (RFS) in the SCC cohort. Ultimately, this work seeks to delineate the immunosuppressive features associated with increasing histological severity and poor outcome, thereby offering evidence to support the development of tailored immunotherapies for cervical precancer and cancer.

## Methods

### Patients and study design

This retrospective study was conducted in accordance with the Declaration of Helsinki and was approved by the Medical Ethics Committee of the Affiliated Hospital of Guizhou Medical University (2022 No. 720). All patients and/or their legal guardians provided informed consent through a process reviewed by this Medical Ethics Committee.

This retrospective study initially screened patients who underwent cervical resection at our institution between January 2014 and December 2021. The sample size was determined by including all consecutive patients who met the predefined strict inclusion and exclusion criteria at our institution. While the sample size is relatively limited due to these rigorous criteria, the exhaustive nature of our institutional sampling helps mitigate selection bias and ensures the biological representativeness of the findings. The inclusion criteria were defined to establish three comparative groups:

1. No cervical lesion (NC) group (n=59): Patients with a postoperative pathological diagnosis of no cervical epithelial lesion and negative preoperative high-risk HPV (HR-HPV) genotyping. These patients underwent surgery (primarily hysterectomy) for other benign gynecological indications, such as symptomatic uterine fibroids or pelvic organ prolapse. This cohort serves as the immunological baseline control.
2. High-grade squamous intraepithelial lesion (HSIL) group (n=52): Patients with a postoperative pathological diagnosis of cervical intraepithelial neoplasia grade II (CIN II) and/or grade III (CIN III). All HSIL diagnoses were histologically confirmed.
3. Squamous cell carcinoma (SCC) group (n=52): Patients with a postoperative pathological diagnosis of cervical squamous cell carcinoma.

#### Rationale for the three-cohort design

This design serves two complementary analytical aims:

- Aim 1 (Descriptive): To characterize and compare the immune microenvironment and systemic inflammatory profiles across the disease continuum—from normal (NC) to pre-invasive (HSIL) to invasive cancer (SCC). The NC group is essential to establish a baseline.
- Aim 2 (Prognostic): To identify factors predictive of postoperative relapse-free survival (RFS) specifically within the SCC cohort. Therefore, survival analysis was performed only on the SCC group n=52.

The exclusion criteria were as follows:

1. Patients who had received any preoperative anticancer therapy, including radiotherapy, chemotherapy, hormonal therapy, or immunosuppressants, or any prior surgical intervention specifically for cervical disease;
2. Pregnant or lactating women;
3. Patients with a concurrent or prior history of other primary malignancies;
4. Patients with hematologic malignancies that could significantly confound inflammatory marker levels;
5. Patients with significant comorbidities, including autoimmune diseases, active infections, or other severe chronic systemic diseases;
6. Patients with incomplete or insufficient key clinical or pathological data (e.g., missing preoperative blood tests, incomplete pathology reports).

Following the application of these criteria, a final cohort of 163 patients was included in the analysis (Supplementary file 1).

Patient information and clinicopathological parameters were extracted from the Hospital Information System, Laboratory Information System, and the Department of Pathology’s database. The collected data encompassed demographic characteristics, laboratory results (including HPV genotyping and complete blood count analysis), and key clinicopathological features such as FIGO stage (2018), tumor diameter, tumor grade, depth of stromal invasion, lymphovascular space invasion, and resection margin status. To ensure data quality, two researchers independently extracted data using standardized forms. Discrepancies were resolved through discussion or, if necessary, consultation with a senior pathologist. A random audit of 10% of cases was performed by a third investigator, demonstrating a data concordance rate of > 99%.

Patients were followed up every 3–6 months for 2 years after the end of surgical treatment, every 6–12 months for 3–5 years, and once a year after 5 years through outpatient consultation or telephone follow-up until December 31, 2022. Relapse-free survival (RFS) was defined as the time from surgery to tumor recurrence, distant metastasis, or death from other causes. Disease recurrence was assessed using physical examinations, high-risk HPV detection, vaginal vault cytology, and computed tomography or magnetic resonance imaging [20, 21].

### Immunohistochemistry (IHC) and scoring of infiltrating lymphocytes

IHC was performed on 3-µm slices of paraffin-embedded tissue blocks. Sections were dried at 80 °C for 1 hour, deparaffinized in xylene, and rehydrated through a graded ethanol series. Antigen retrieval was achieved using sodium citrate buffer or EDTA (Solarbio LifeScience, Beijing, C1032 and C1034) as per the manufacturer’s instructions. Endogenous peroxidase activity was quenched with 3% H_2_O_2_. Slides were blocked with normal goat serum (Solarbio LifeScience, Beijing, SL038) and incubated overnight at 4 °C with the corresponding primary antibody [see Additional file 3). Afterward, slides were incubated for 1 hour at 37 °C with horseradish peroxidase (HRP)-conjugated secondary antibody (Zhongshan Jinqiao, Beijing, PV-6000), and target lymphocytes were detected using a 3,3’ diaminobenzidine substrate (Zhongshan Jinqiao, Beijing, ZLI-9018). Images were captured with a Leica DM6 M LIBS microscope. Two experienced pathologists, blinded to the case information, independently analyzed the IHC results. Staining intensity and the percentage of stained cells were semi-quantitatively converted into a histoscore (H-score) on a scale of 0–300 [22, 23]. Representative images of TIICs in the tumor/lesion center (TC) and the tumor stroma (TS) [24, 25] are shown in Additional file 4.

### Multiplex Immunofluorescence (mIF)

TG 7 color fluorescent staining kits (TissueGnostics, Vienna, Austria, TGFP750) were used for mIF staining. Primary antibodies [Additional file 3) were incubated at 37 °C for 1 h, followed by HRP-conjugated secondary antibodies and tyramine signal amplification (TSA). After each TSA procedure, the slides were microwave-heated to expose the antigens. Cell nuclei were counterstained with TG SN470 and mounted with a mounting medium (Panovue, Beijing). Images were acquired using Tissue FAXS SL scanning and analyzed with Strata Quest software (Tissue Gnostics, Vienna, Austria). The percentage of cells positive for dual and triple markers was calculated as the number of positive cells divided by the total number of CD4 + cells.

### SIR markers

Peripheral venous blood samples were collected within 2 weeks preoperatively for routine tests. An absolute neutrophil count (ANC, cells/µL), absolute lymphocyte count (ALC, cells/µL), absolute monocyte count (AMC, cells/µL), and absolute platelet count (PLT, cells/µL) were obtained using the Sysmex XN-9000 hematology analyzer (SYSMEX, Kobe, Japan). The ratios of lymphocyte-to-monocyte (LMR = ALC/AMC), neutrophil-to-lymphocyte (NLR = ANC/ALC), and platelet-to-lymphocyte (PLR = PLT/ALC) were also calculated [26].

### Statistical analyses

Statistical analyses were performed using OriginPro (version 9.7) and GraphPad Prism (version8.0). A P value < 0.05 was considered significant. After assessing variance homogeneity with Levene‘s test, group comparisons (NC, HSIL, SCC) were made using one-way ANOVA or the Kruskal-Wallis test, with post-hoc least significant difference tests. Inter-group comparisons used the independent sample t-test, and correlations were assessed by Spearman’s rank coefficient.

To identify factors associated with histologic stage advancement (e.g., HSIL vs. NC; SCC vs. HSIL), univariate and multivariate binary logistic regression analyses were employed.

For prognostic analysis within the SCC cohort, relapse-free survival (RFS) was evaluated. Univariate Kaplan–Meier analysis (log-rank test) was followed by a multivariate Cox regression model. Crucially, this Cox model included key clinical prognostic factors (FIGO stage, tumor size, depth of invasion, LVSI) in addition to TIICs and SIR markers to identify independent predictors. Receiver operating characteristic (ROC) curve analysis (GraphPad Prism) and principal component analysis (PCA, OriginPro) were used for diagnostic evaluation and data visualization, respectively.

## Results

### Evaluation of tiics infiltration and SIR across different histological stages of cervical lesions

**Fig. 1:**
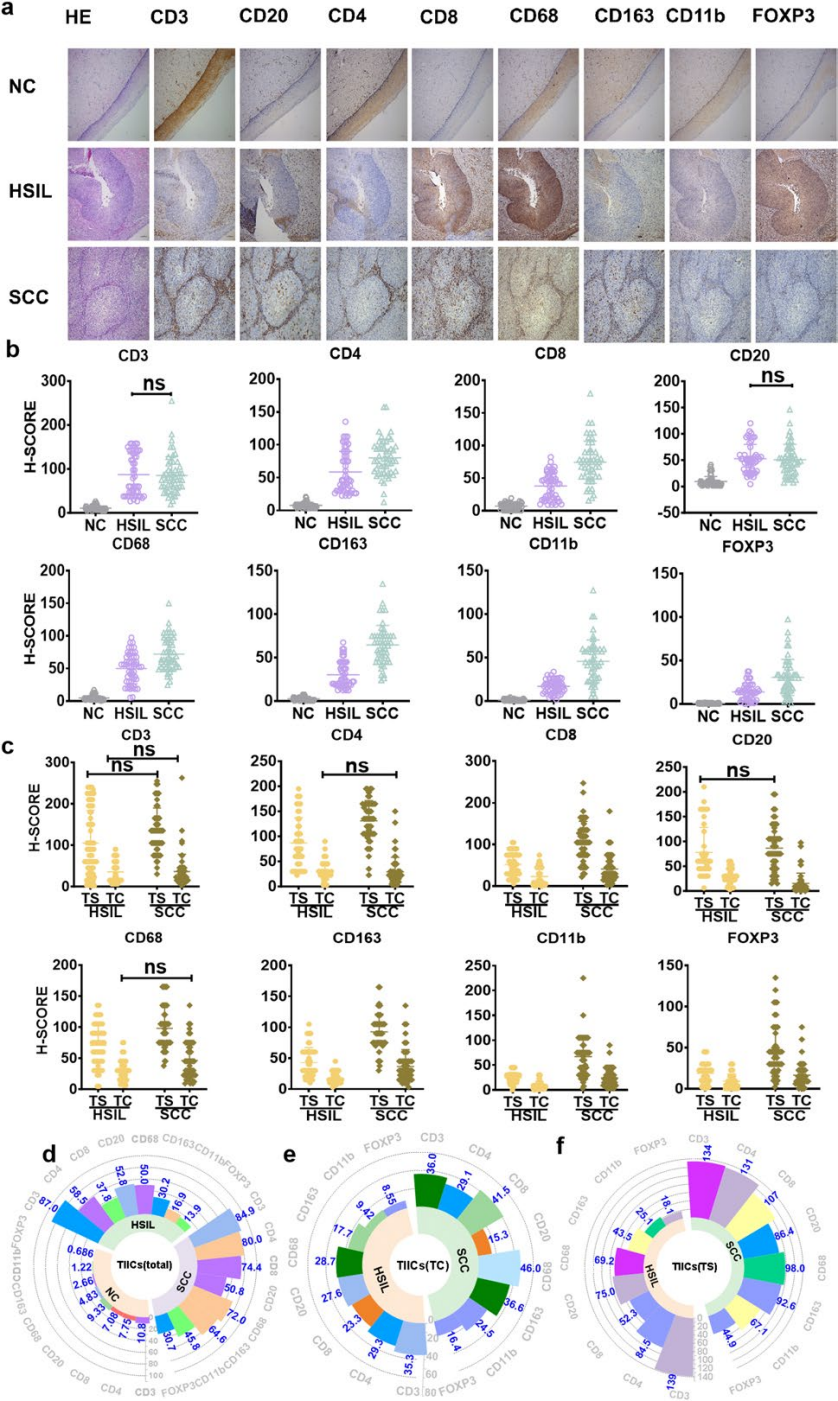
TIICs infiltration in NC, HSIL, and SCC groups. **a** Representative images of immunohistochemistry staining of infiltrating lymphocytes in cervical tissue specimens from NC, HSIL, and SCC populations (×100, scale bar: 100 μm). All TIICs are located on the surface of the cell membrane except for FOXP3, which is present in the nucleus. **b** and **d** Corresponding scatter dot and radial bar plots for the overall H-score, respectively. **c**, **e**, and **f** Scatter dot plots and radial bar plots for H-scores of tTIICs and sTIICs in HSIL and SCC. All *P*-values are < 0.05 unless marked with ‘‘ns’’ (*P* ≥ 0.05, not significant). NC no cervical epithelial lesions, HSIL high-grade squamous intraepithelial lesion, SCC squamous cell carcinoma of the cervix, TC tumor/lesion center, TS tumor stroma, s tumor stroma, t tumor/lesion center

TIICs infiltration was rare in NC but evident in HSIL and increased further in SCC (Fig. 1a). Statistically significant inter-group differences were observed in TIICs for all types except CD3 + T and CD20 + B cells (*P* < 0.05, Fig. 1b). The differences in TIICs infiltration were more pronounced between NC and HSIL than between HSIL and SCC. In HSIL and SCC tissues, sTIICs and tTIICs showed significantly higher levels of infiltration in the TS than in the TC (Fig. 1c). The proportion of immunosuppressive cells (CD163+, CD11b+, and FOXP3 + Tregs cells) increased in the HSIL group and was even more pronounced in the SCC group. (Fig. 1d–f), suggesting that HSIL represents an initial stage of immunosuppression in cervical lesions.

**Fig. 2:**
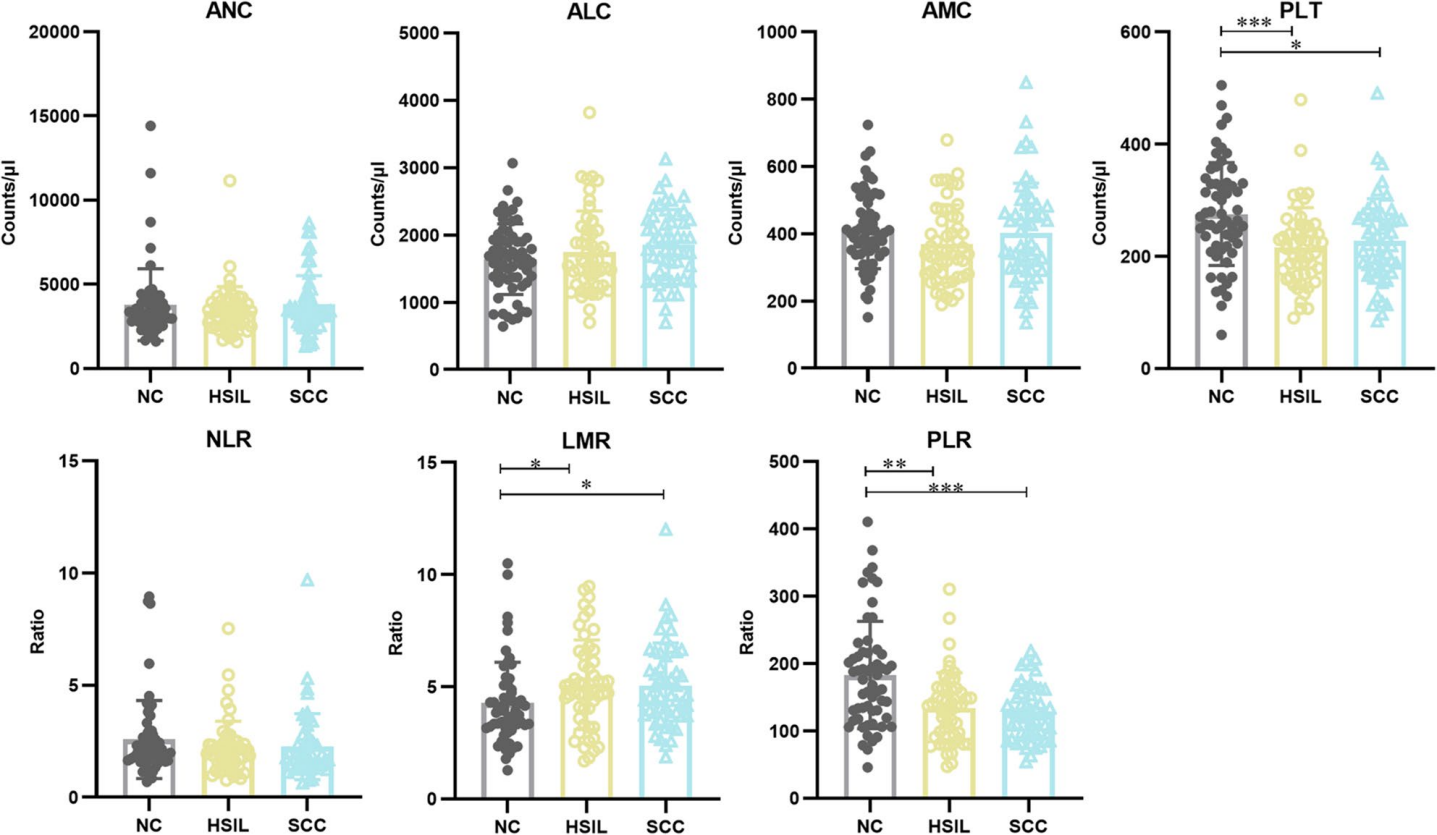
SIR markers in peripheral blood of NC, HSIL, and SCC populations. ANC absolute neutrophil count, ALC absolute lymphocyte count, AMC absolute monocyte count, NLR neutrophil-to-monocyte ratio, LMR lymphocyte-to-monocyte ratio, PLR platelet-to-lymphocyte ratio, NC no cervical epithelial lesions, HSIL high-grade squamous intraepithelial lesion, SCC squamous cell carcinoma of the cervix, SIR systemic inflammation response. *P*-values are obtained via the Kruskal**–**Wallis test and Dunn’s multiple tests (*P < 0.05; **P < 0.01; ***P < 0.001)

Among the preoperative SIR markers, PLT was significantly lower in HSIL and SCC than in NC, primarily contributing to PLR reduction due to a minor increase in ALC. However, the moderate decrease in AMC and the minor increase in ALC from NC to HSIL and SCC resulted in a significant increase in LMR in the lesion groups (Fig. 2).

**Fig. 3:**
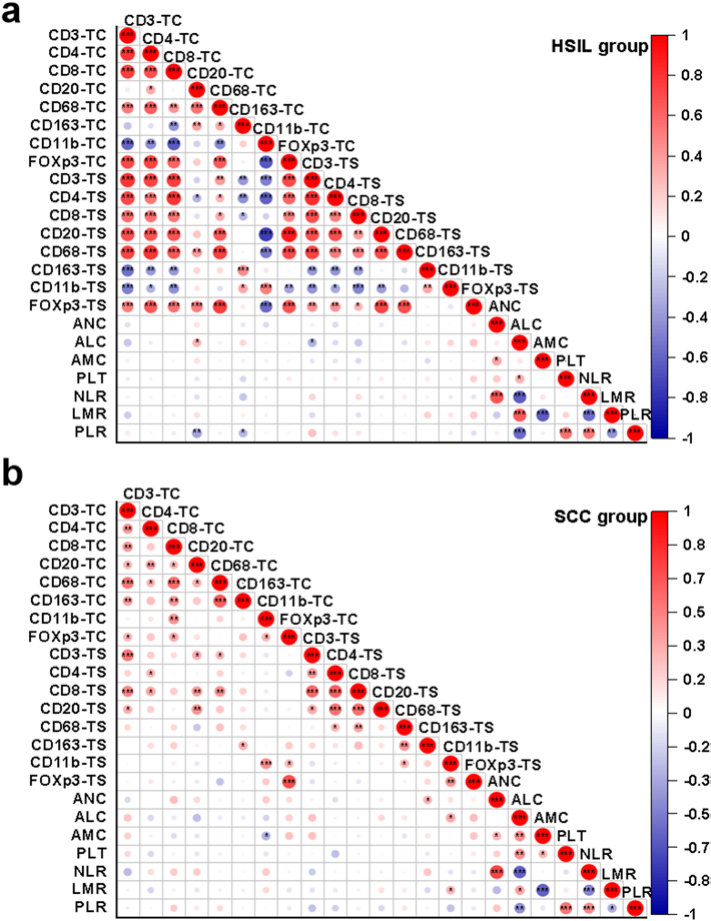
Correlations between TIICs and SIR in HSIL and SCC. **a** and **b** Correlations of TIICs (TC and TS areas) and SIR in the HSIL and SCC groups. Red indicates a positive correlation, white represents no correlation, and blue represents a negative correlation. The darker the color and the bigger the circle, the higher the correlation between the two variables. Significant correlations are marked with asterisks (*P < 0.05; **P < 0.01; ***P < 0.001). HSIL high-grade squamous intraepithelial lesion, SCC squamous cell carcinoma of the cervix, SIR systemic inflammation response, TIICs tumor-infiltrating immune cells, TC tumor/lesion center, TS tumor stroma, ANC absolute neutrophil count, ALC absolute lymphocyte count, AMC absolute monocyte count, NLR neutrophil-to-monocyte ratio, LMR lymphocyte-to-monocyte ratio, PLR platelet-to-lymphocyte ratio

In both TC and TS of HSIL, cells that may drive tumor immunosuppression, including CD163 + M2 macrophages and CD11b + myeloid cells were negatively correlated with other immune cells. Most TIICs in the TS corresponded to those in the TC (*P* < 0.001), except for CD20 + cells (Fig. 3a). In contrast, the correlation of TIICs was disrupted in the SCC group (Fig. 3b), and SIR was not significantly correlated with TIICs invasion in HSIL and SCC.

### Factors associated with cervical lesion severity

**Fig. 4:**
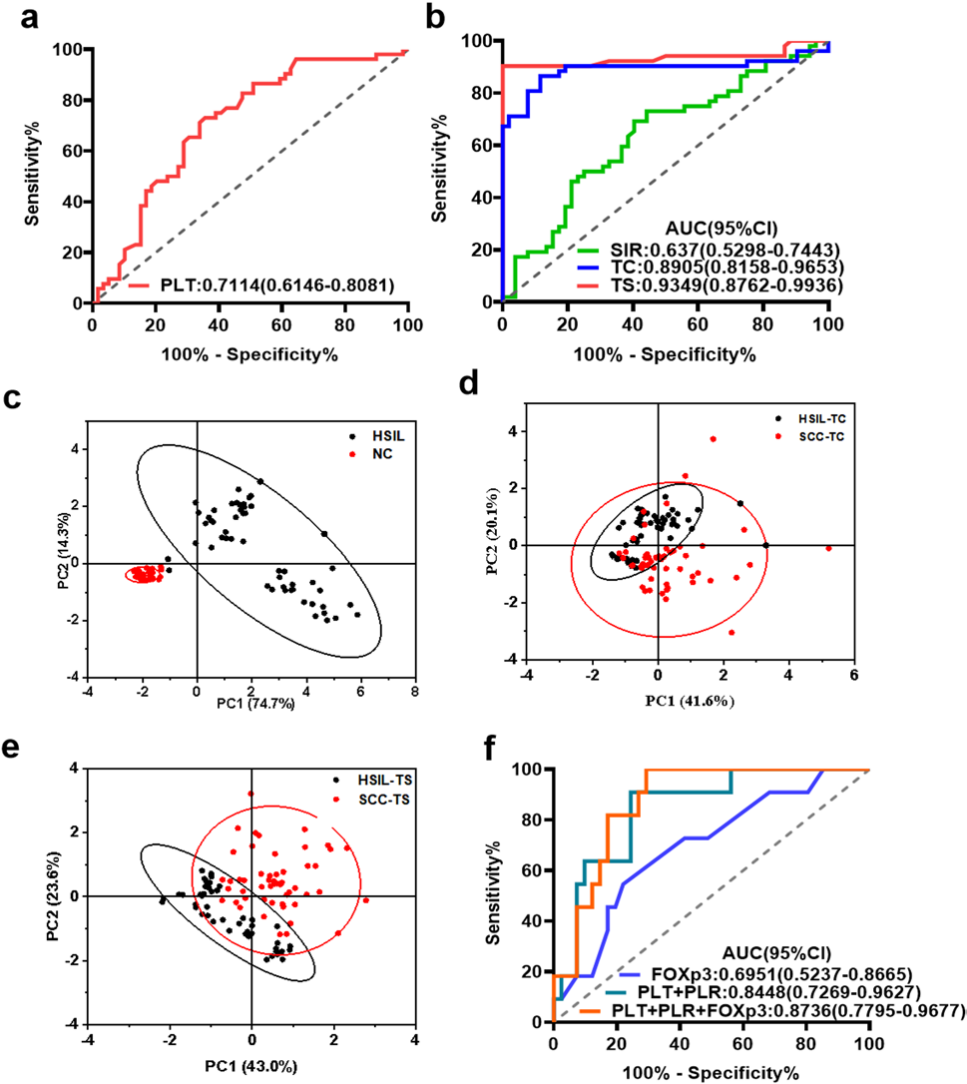
Diagnostic efficiency of TIICs and SIR in distinguishing histological stages and recurrence of cervical cancer. **a** and **b** ROC curve of PLT, SIR (ANC + PLT + NLR + LMR + PLR), TC (CD3 + CD8 + CD20 + CD163 + FOXP3), and TS (CD8 + CD11b) across the spectrum from NC to SCC. **c**–**e** PCA results for TIICs in the NC, HSIL, and SCC groups. Each symbol denotes an individual patient, and 95% confidence ellipses are drawn. **f** ROC curve of FOXp3, PLT, and PLR in SCC recurrence. ROC Receiver operating characteristic curve, PCA principal component analysis, NC no cervical epithelial lesions, HSIL high-grade squamous intraepithelial lesion, SCC squamous cell carcinoma of the cervix, SIR systemic inflammation response, TIICs tumor-infiltrating immune cells, TC tumor/lesion center, TS tumor stroma, PLR platelet-to-lymphocyte ratio, ANC absolute neutrophil count, LMR lymphocyte-to-monocyte ratio, NLR neutrophil-to-lymphocyte, AUC area under the curve

No TIICs variables were significantly associated with the presence of HSIL compared to NC (*P* > 0.05; Table 1), although PLT was identified as a significant indicator. In distinguishing SCC from HSIL, tFOXP3+, tCD163+, sCD8+, and sCD11b + cells, along with PLT, PLR, ANC, NLR, and LMR, were identified as significant associated factors. Receiver operating characteristic (ROC) analysis revealed that, sTIICs had greater diagnostic efficacy for distinguishing SCC from HSIL than tTIICs (Fig. 4a and b). Principal component analysis revealed that TIICs infiltration clearly distinguished cases across different cervical lesion stages, with sTIICs showing more pronounced discrimination ability (Fig. 4c and d).

**Table 1:** Univariate and multivariate logistic regression analysis of factors associated with cervical lesion progression

	Variable	Univariate			Multivariate			
		95% CI		*P*	OR	95% CI		*P*
NC(overall)vs.HSIL	CD3	0.000		0.996	139,000,000	0.000		0.998
	CD4	0.000		0.996	1423.426	0.000		1.000
	CD8	25.715	409.891	0.000*	97359.960	0.000		1.000
	CD20	39.230	2694.554	0.000*	4,778,833	0.000		0.998
	CD68	0.000		0.997	0.613	0.000		1.000
	CD163	0.000		0.996	13,229,699	0.000		1.000
	CD11b	180.376	48508.508	0.000*	0.462	0.000		1.000
	FOXP3	75.258	6436.750	0.000*	1.362	0.000		1.000
	ANC	0.743	3.896	0.208	0.900	0.286	2.831	0.858
	ALC	0.041	0.941	0.042*	0.414	0.062	2.775	0.364
	AMC	1.319	6.524	0.008*	2.022	0.672	6.082	0.210
	PLT	2.180	11.064	0.000*	3.811	1.315	11.041	0.014*
	NLR	0.993	6.728	0.052	0.925	0.252	3.399	0.907
	LMR	0.088	0.441	0.000*	0.362	0.116	1.127	0.080
	PLR	2.959	24.362	0.000*	2.673	0.654	10.924	0.171
HSILvsSCC	tCD3	0.142	1.040	0.060	0.105	0.011	0.994	0.049*
	sCD3	0.145	0.851	0.021*	0.241	0.007	8.440	0.433
	tCD4	0.203	0.996	0.049*	0.723	0.102	5.120	0.746
	sCD4	4.036	26.741	0.000*	9,368,757,542	0.000		0.995
	tCD8	2.370	13.838	0.000*	10.842	1.217	96.607	0.033*
	sCD8	8.751	182.845	0.000*	60.068	1.036	3483.695	0.048*
	tCD20	0.035	0.221	0.000*	0.056	0.007	0.465	0.008*
	sCD20	1.724	8.774	0.001*	0.231	0.005	10.263	0.449
	tCD68	1.434	7.749	0.005*	1.644	0.220	12.292	0.628
	sCD68	2.099	14.464	0.001*	0.344	0.015	7.997	0.506
	tCD163	2.605	14.213	0.000*	8.491	1.365	52.811	0.022*
	sCD163	15.369	158.052	0.000*	2,374,192,715	0.000		0.995
	tCD11b	4.532	28.679	0.000*	5.676	0.909	35.449	0.063
	sCD11b	8.790	74.330	0.000*	65.405	1.220	3506.536	0.040*
	tFOXP3	1.804	9.794	0.001*	12.486	1.337	116.621	0.027*
	sFOXP3	3.390	19.580	0.000 *	18.818	0.837	423.155	0.065
	ANC	0.211	1.218	0.129	0.301	0.095	0.956	0.042*
	ALC	0.345	1.959	0.659	3.287	0.910	11.872	0.069
	AMC	0.215	1.070	0.073	1.632	0.520	5.117	0.401
	PLT	0.158	0.990	0.048*	0.146	0.039	0.543	0.004*
	NLR	0.855	4.075	0.117	3.602	1.099	11.799	0.034*
	LMR	0.855	4.075	0.117	5.264	1.551	17.871	0.008*
	PLR	0.792	3.769	0.169	4.045	1.171	13.972	0.027*

*TIICs* Tumor-infiltrating immune cells, *SIR* Systemic inflammatory response, *NC* No cervical epithelial lesions, *HSIL* High-grade squamous intraepithelial lesions, *SCC* Squamous cell carcinoma of the cervix, *ANC* Absolute neutrophil count, *ALC* Absolute lymphocyte count, *AMC* Absolute monocyte count, *PLT* Absolute platelet count, *NLR* Ratios of neutrophil-to-lymphocyte, *LMR* Ratios of lymphocyte-to-monocyte, *PLR* Ratios of platelet-to-lymphocyte, *95% CI*, 95% Confidence interval, *OR* Odds ratio, *s* tumor stroma, *t* tumor/lesion center

*P < 0.05

### Prognostic risk factors in SCC

**Fig. 5:**
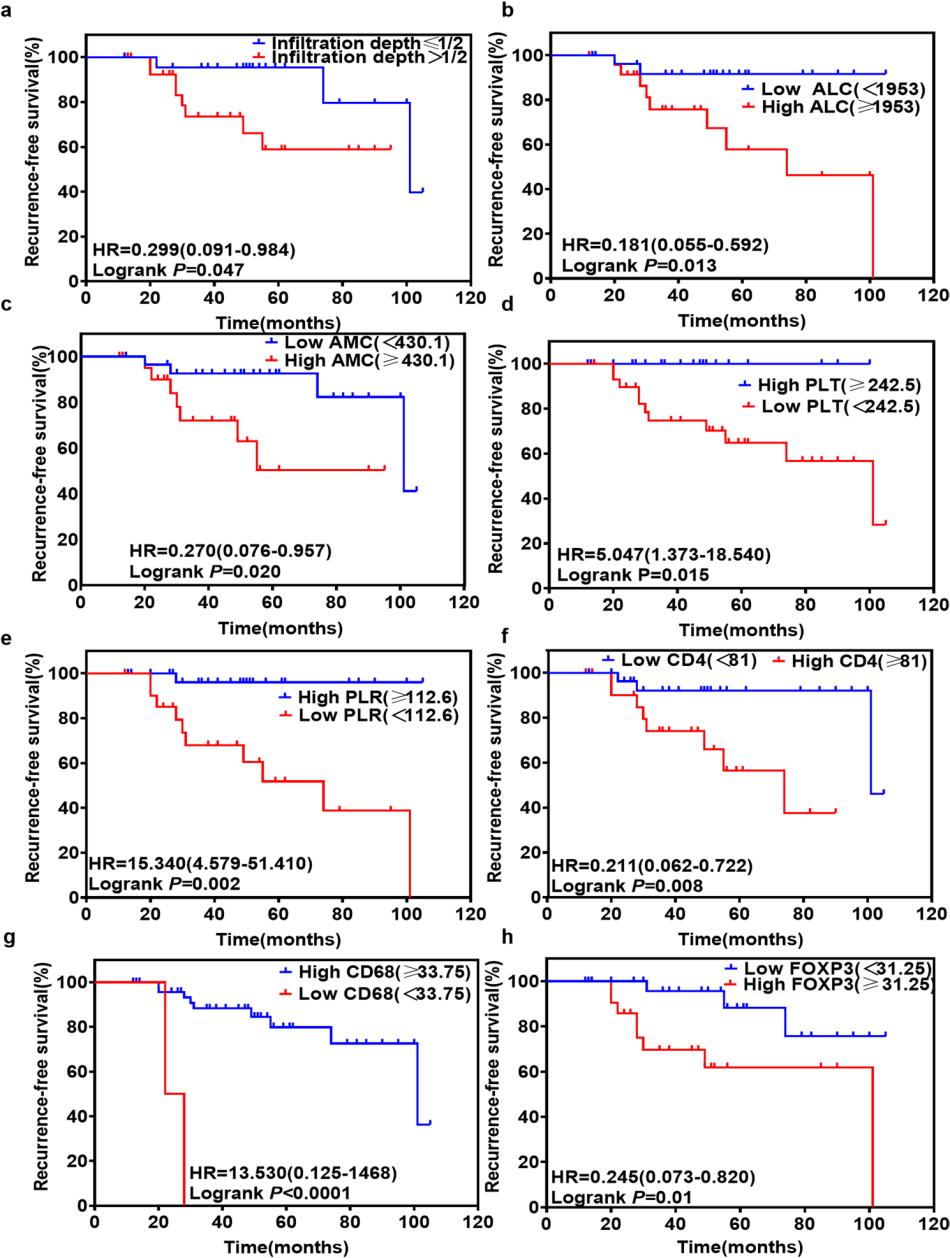
Kaplan–Meier survival curves of clinical factors, SIR, and TIICs with statistical significance for RFS. **a** Tumor infiltration depth, **b** ALC, **c** AMC, **d** PLT, **e** PLR, **f** CD4^+^ T cells, **g** CD68^+^ cells, and **h** FOXP3^+^ Tregs are significant predictors of RFS in patients with SCC. SIR systemic inflammation response, TIICs tumor-infiltrating immune cells, PLT platelets, PLR platelet-to-lymphocyte ratio, ALC absolute lymphocyte count, AMC absolute monocyte count, SCC squamous cell carcinoma of the cervix, RFS Relapse-free survival

In 52 patients with cervical cancer followed for an average of 47.5 months (range: 12–105 months), 21.15% (11/52) experienced relapse, while 78.85% (41/52) had no recurrence. Kaplan–Meier analysis revealed lower PLT, PLR, and CD68 + cells, along with higher depth of invasion, ALC, AMC, CD4 + T cells, and FOXP3 + Tregs, as poor prognostic factors for RFS in SCC (Fig. 5 and Additional file 5). Cox proportional hazards regression indicated that FOXP3 + Tregs were a significant risk factor for SCC prognosis (Table 2). ROC curve analysis demonstrated that combined detection of FOXP3, PLT, and PLR outperformed single tests in predicting SCC recurrence (Fig. 4f).

**Table 2:** Cox multivariate regression analysis of indicators screened from univariate analysis for RFS in patients with SCC

	Variable(cutoff)		Multivariate		
		HR	95% CI		*P*
SIR	ALC (1953)	4.935	0.926	26.301	0.062
	AMC (430.1)	3.118	0.645	15.080	0.157
	PLT (242.5)	0.000	0.000	1.35E + 139	0.945
	PLR (112.6)	0.463	0.044	4.907	0.523
TIICs (Overall)	CD4 (81.00)	662897.645	0.000	1.99E + 136	0.930
	CD68 (33.75)	0.000	0.000	1.06E + 124	0.923
	FOXP3 (31.25)	8.723	1.017	74.812	0.048*

*RFS* Relapse-free survival, *SCC* Squamous cell carcinoma of the cervix, *SIR* Systemic inflammatory response, *TIICs* Tumor-infiltrating lymphocytes, *ALC* Absolute lymphocyte count, *AMC* Absolute monocyte count, *PLT* Absolute platelet count, *PLR* Platelet-to-lymphocyte ratio, *HR* Hazard Ratio, *95% CI 95%* Confidence interval

^*^P < 0.05

### Evaluation of exhausted CD4⁺ T‑cell infiltration in cervical lesion across different histological stages

**Fig. 6:**
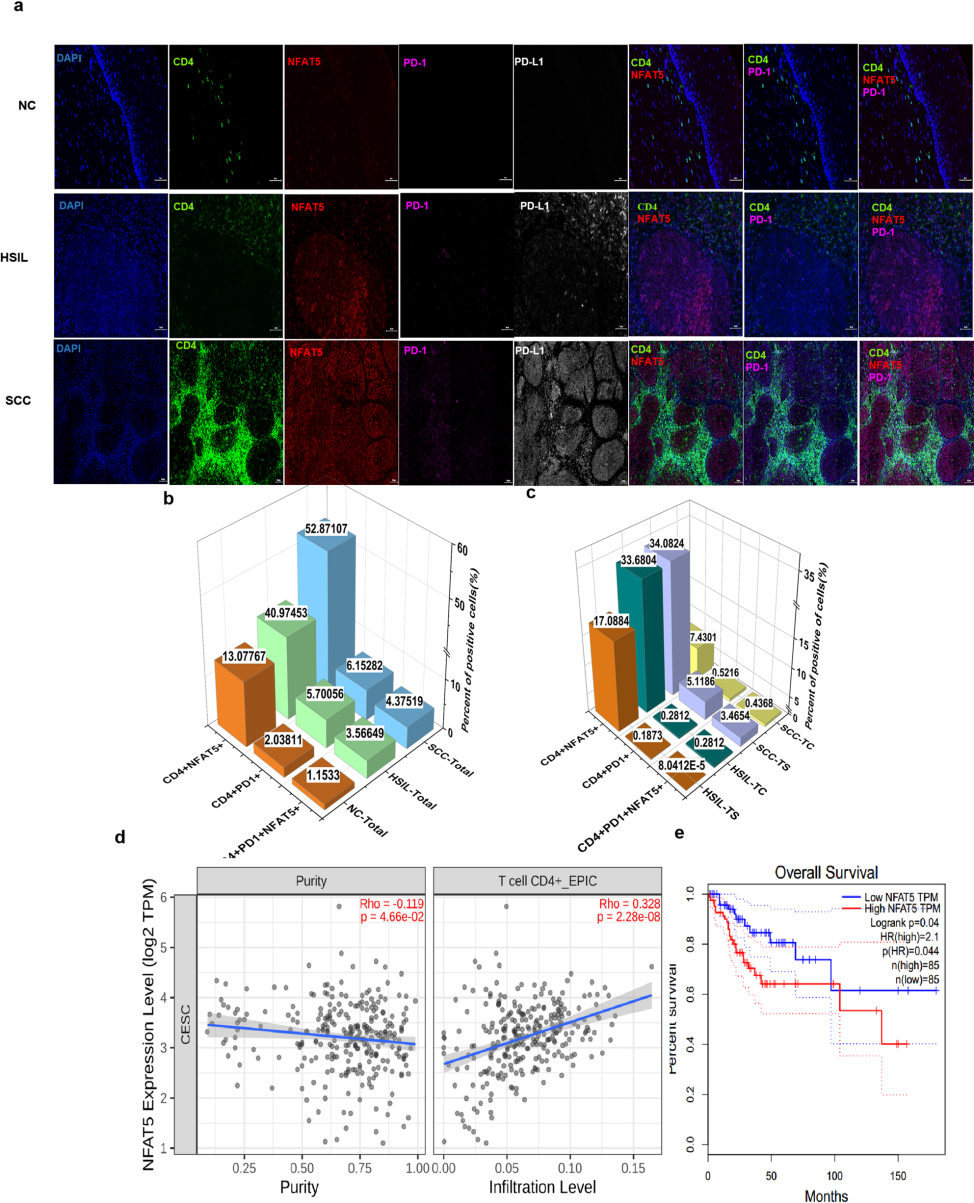
Expressions of exhausted CD4⁺ T‑cell in NC, HSIL, and SCC groups. **a** Representative images of multiplex immunofluorescence staining of CD4, NFAT5, PD-1, and PD-L1 in cervical tissue specimens from the NC, HSIL, and SCC populations. Nucleus (blue), CD4 (green), NFAT5 (red), PD-1 (pink), and PD-L1 (white) (white bars = 50 μm). CD4^+^ PD-1, CD4^+^ NFAT5^+^, and CD4^+^ NFAT5^+^ PD-1^+^ cells in total region infiltration (**b**) and TC and IM infiltration (**c**) from the NC, HSIL, and SCC groups. **d** The correlation between CD4^+^ T-cell levels and NFAT5 expression analyzed using the TIMER3.0 database. **e** Overall survival based on NFAT5 expression analyzed using the GEPIA database. NC no cervical epithelial lesions, HSIL high-grade squamous intraepithelial lesion, SCC squamous cell carcinoma of the cervix, TC tumor/lesion center, TS tumor stroma, PD-1 programmed cell death protein 1, PD-L1 programmed death-ligand 1, NFAT5 nuclear factor of activated T cells. 5

High CD4 + T-cell infiltration is associated with a poorer prognosis in patients with SCC, suggesting the potential involvement of CD4 + T-cell exhaustion in pathological advancement. As a critical transcription factor in T cell exhaustion, NFAT5 expression in cervical cancer was positively correlated with CD4⁺ T cell infiltration(Fig. 6a), and patients with high NFAT5 expression exhibited a poorer prognosis. (Fig. 6b) The proportions of CD4 + PD-1 + NFAT5 + TIICs and PD-L1 + cells increased across the histological stages from NC to SCC (Fig. 6c and d). In SCC, sCD4 + PD-1 + NFAT5 + TIICs were more prevalent than tCD4 + PD-1 + NFAT5 + TIICs, indicating greater exhaustion of CD4 + T cells in the TS (Fig. 6e).

## Discussion

The development and carcinogenesis of tumors are closely linked to dynamic interactions with the immune system. Cellular immune responses play a critical role in HPV-associated cervical cancer [27]. In this study, we observed that the pathological advancement of cervical lesions was accompanied by an increase in the infiltration of TIICs, particularly immunosuppressive subsets such as CD163⁺, CD11b⁺, and FOXP3⁺ cells. Notably, immune cell density is significantly higher in the tumor stroma than within tumor/lesion center. Furthermore, the infiltration of FOXP3⁺ Treg cells and PLT are identified as key factors associated with both histological severity and patient prognosis. Additionally, exhausted CD4⁺ T cell infiltration increases with increasing lesion severity and demonstrates spatial distribution heterogeneity across different stages of disease progression.

Our findings revealed a significant increase in TIICs from NC to HSIL, and the number of immunosuppressive cells (CD68+, CD163+, CD11b+, and FOXP3 + cells) continued to rise from HSIL to SCC. TIICs subsets (CD3 + and FOXP3 + cells) are elevated in SCC and associated with risk stratification in cervical cancer [28]. Conversely, our study found that TIICs were significantly elevated in HSIL compared with those in NC, a finding less emphasized in prior research [29]. HSIL has a high potential for malignant transformation, and conventional surgical treatments can lead to complications such as hemorrhage, infection, and increased risk of premature birth in subsequent pregnancies [30]. Spontaneous regression of HSIL is associated with higher infiltration of CD8 + and CD4 + T cells than persistent lesions [31]. Pre-existing CD4 + T cells correlate strongly with complete responses to immunotherapy in HSIL [32]. It is noteworthy that while some studies suggest spontaneous regression of HSIL is associated with higher infiltration of functional CD8 + and CD4 + T cells, our results showed a continuous increase in TIICs density as lesions progressed to SCC. This apparent discrepancy can be reconciled by considering the functional quality of the immune infiltrate. In regressive lesions, TIICs are likely effector cells performing active immunosurveillance. However, our multiplex immunofluorescence data revealed that the increased TIICs in SCC, particularly the CD4 + subset, significantly co-express exhaustion markers such as PD-1 and NFAT5. This suggests that the high-density infiltration in advanced lesions represents an accumulation of exhausted and ineffective immune cells, which fails to halt tumor progression, leading to a poorer prognosis despite higher cell counts.

Meanwhile, we found that the TS region exhibited significantly higher infiltration of immunosuppressive cells than the TC region in both HSIL and SCC groups. The TS region may serve as a crucial source of cells that exert either antitumor or immunosuppressive effects and could offer a more sensitive and accurate region for prognostic evaluation than intraepithelial TIICs, as suggested by studies on early- stage and advanced-stage cervical cancer [33, 34]. These findings raise questions about the distinct significance of the TS in tumor biology and the role of crosstalk between the TC and TS in malignant transformation and metastasis.

Across different histological stages, tCD3 + T, tCD20 + B, tCD8+, and tCD163 + cells, along with an increase in immunosuppressive cells such as sCD11b + and sFOXP3 + Tregs, are associated with the presence of SCC. Additionally, sTIICs demonstrate greater diagnostic efficacy in distinguishing SCC from HSIL, suggesting that enhanced immunosuppression contributes to pathological advancement. The TS may be a more critical region for tumor immune response than the TC. As a reactive tumor stroma at the invasive front, the TS is rich in immune cells and peritumoral vasculature. It influences extracellular matrix remodeling and regulates immune cells, including CD4 + Th cells, CD8 + cytotoxic T cells, Tregs, and macrophages, by secreting matrix proteases, growth factors, and cytokines [35]. In colorectal cancer liver metastases, TIICs densities in the TS predicted chemotherapy response with 100% specificity and 79% sensitivity [36]. Thus, our findings on TIICs infiltration patterns in HSIL and SCC within the TS may aid in distinguishing lesion stages and predicting prognosis.

An infiltration depth greater than half was associated with a higher postoperative recurrence rate, while high FOXP3 + Treg infiltration predicted postoperative recurrence in cervical cancer. Elevated FOXP3 + Treg levels correlate with poor cancer prognosis [37, 38], consistent with our findings.Our findings are further corroborated by a recent meta-analysis of 21 studies [39], which demonstrated that elevated Treg infiltration is significantly associated with shorter survival and more advanced clinical stages across various cohorts, reinforcing the role of Tregs as reliable prognostic determinants in cervical cancer. Meanwhile, our patients with high CD4 + T-cell infiltration had shorter RFS. This contrasts with Wang et al. [40], who reported that activated memory CD4 + T cells correlated independently with favorable overall survival (OS). The discrepancy may be attributed to their precise identification of activated memory CD4 + T cells via bioinformatics, whereas our marker (CD4) broadly identifies all CD4 + T-cell subsets. CD4 + T cells can differentiate into Th cells and Tregs, where Th cells assist CD8 + T cells in antitumor immunity, and Tregs promote immune suppression and tumor progression. Although our results indicate that FOXP3 + Tregs (which express CD4) increase during cervical pathological evolution, their numbers were significantly lower than those of CD4 + T cells, suggesting that most CD4 + T cells still differentiate into Th cells. Combined survival analysis reveals that CD4 + T cells may exhibit functional impairment in advanced lesions, warranting further confirmation.

We found that PLT reduction was the most significant change among SIR factors, plateauing in HSIL. PLT is a significant indicator associated with the histological advancement of cervical epithelium. Lower PLT and calculated PLR are associated with shorter RFS. Notably, FOXP3 + Treg infiltration combined with PLT and PLR more accurately predicts cervical cancer recurrence, differing from previous studies. Peripheral blood SIR factors, including PLT, are easily accessible, reproducible, cost-effective, and effectively identify patients at high risk for poor outcomes and mortality [41, 42], consistent with our findings. Women with cervical bleeding are at higher risk for abnormal cervical smears and cervical cancer [43]. Patients with HSIL may also experience mild bleeding, contributing to lower PLT in HSIL and SCC than that in NC. Viral infections, such as human immunodeficiency virus, Epstein–Barr virus, and hepatitis C virus infections, can affect platelet count production and destruction [44]. Therefore, we speculate that the reduction in platelets in HSIL and SCC is due to HR-HPV infection and mild bleeding. PLT combined with FOXP3 + TIICs can distinguish lesion stages and prognosis of cervical lesions, making it a clinically useful biomarker.

To further explain the high CD4 + T-cell infiltration and poor prognosis in cervical cancer, we focus on T-cell exhaustion, which plays a crucial role in chronic infections and cancers where sustained antigen stimulation can induce exhaustion and affect pathological advancement and prognosis [45]. We found that the proportion of CD4 + PD-1 + cells in the HSIL and SCC groups was significantly higher than that in the NC group. Classical exhaustion markers, such as PD-1, are present on CD4 + T cells [46, 47], consistent with our results in cervical cancer. Analysis in melanoma lymph nodes has shown that NFAT5 is highly expressed in infiltrating CD8^+^ T cells [48]. Tillé et al. showed that NFAT5 may promote the attenuation of T-cell-related effectors and activation signals to regulate T-cell exhaustion [49]. The binding of NFAT to the PD-1 promoter induces the expression of Pdcd1 (encoding PD-1) [50, 51]. In our study, the infiltration of CD4 + NFAT5 + and CD4 + PD-1 + NFAT5 + cells increased with histological severity. In the HSIL group, CD4 + PD-1+, CD4 + NFAT5 + T, and CD4 + PD-1 + NFAT5 + T cells were more numerous in the TC region than in the TS region, although the overall count in the TS region exceeded that in the TC region. These findings indicate a significant difference in exhausted T-cell infiltration between precancerous lesions and cervical cancer. PD-L1 levels are elevated in advanced lesions, supporting the role of the PD-1 and PD-L1 pathway in T-cell exhaustion. While exhausted CD4 + T cells have been documented in other cancers, this is the first report of such in cervical cancer. However, further studies are needed to determine the underlying causes of exhausted CD4 + T cells and their role in pathological advancement.

## Limitations

This study has several potential limitations. First, as a single-center study, less than 10% of over 2,000 cervical sections collected qualified for inclusion after strict screening, which may have introduced bias due to the small sample size. However, our stringent exclusion criteria minimized other variables, such as prior exposure to radiation, chemotherapy, hormone therapy, immunosuppressants, primary surgical treatments (including allergy-related and tumor surgeries), other viral infections beyond HPV, autoimmune diseases, and the effects of chronic diseases on immune function. Consequently, this study provides an objective perspective on TIICs infiltration from persistent HPV infection to the development of cervical cancer. Second, this study is retrospective, and further prospective studies are needed to confirm these findings. However, our results suggest distinct characteristics of TIICs infiltration in HSIL, indicating that intervention in immunosuppressive TIICs infiltration may reduce the long-term need for surgical treatment. Third, while we observed changes in TIICs counts, our study lacks data on TIICs subset functionality. Preliminary observations show exhausted CD4 + T cells in HSIL and SCC tissues, which may partially explain the relationship between CD4 + T cell counts and survival prognosis. Further research is needed to assess functional changes in other TIICs subsets. Finally, due to the cross-sectional nature of our study, we can identify immune features that differ between established HSIL and SCC but cannot definitively establish them as causative predictive factors for event of progression. A longitudinal study tracking HSIL patients over time is required to validate which immune profiles truly predict malignant transformation. Additionally, the identification of specific immune subsets was limited by the choice of markers. For example, CD11b, used as a marker for immunosuppressive myeloid cells, is not exclusive to MDSCs but is expressed broadly across the myeloid lineage. Future studies with more extensive phenotypic panels are needed to precisely delineate these populations.

## Conclusions

In conclusion, our findings demonstrate that TIICs infiltration correlates with the histological severity of cervical lesions, particularly within the TS region. Specific TIICs (tFOXP3+, tCD163+, sCD8+, and sCD11b + cells) and SIR markers (PLT, PLR, ANC, NLR, and LMR) may serve as valuable biomarkers for distinguishing SCC from HSIL. Routine monitoring of TIICs and SIR following surgical treatment for HSIL may provide insights into the immunological landscape of cervical lesion advancement. FOXP3 + Tregs, when combined with the SIR factor PLT in peripheral blood, could help predict SCC recurrence. For patients with SCC in postoperative follow-up, monitoring FOXP3 + Tregs along with PLT levels may be particularly useful for assessing recurrence risk. Additionally, the progressive increase in exhausted CD4 + T cells in HSIL and SCC suggests the need to monitor both TIICs functionality and quantity.

## Supplementary Information

Supplementary Material 1. A flow diagram to summarize the screening process of the study subjects.

Supplementary Material 2. Clinical characteristics of study subjects, n=163.

Supplementary Material 3. Antibodies used for immunohistochemistry (IHC) and multiplex immunofluorescence (mIF).

Supplementary Material 4. Representative images to demonstrate the delineation of the TC and TS areas in HSIL (a) and SCC (b) tissue samples.

Supplementary Material 5. Univariate Kaplan-Meier analysis of Clinicopathologic features, SIR and TIICs (Overall) in SCC patients.

## Data Availability

The original contributions presented in this study are included in the article and supplementary materials. For further inquiries, please contact the corresponding author.

## Abbreviations

ALC: Absolute lymphocyte count
AMC: Absolute monocyte count
ANC: Absolute neutrophil count
HR-HPV: High-risk types of human papillomavirus
HRP: Horseradish peroxidase
H-score: Histochemistry Score
HSIL: High-grade squamous intraepithelial lesions
IHC: Immunohistochemistry
LMR: Lymphocyte-to-monocyte ratio
MDSCs: Myeloid-derived suppressor cells
mIF: Multiplex immunofluorescence
NFAT5: Nuclear factor of activated T cells 5
NLR: Neutrophil-to-lymphocyte
NMR: Neutrophil-to-monocyte ratio
PD-1: Programmed cell death protein 1
PLR: Platelet-to-lymphocyte ratio
PLT: Platelets
ROC: Receiver operating characteristic curve
RFS: Relapse-free survival
SCC: Squamous cell carcinoma of the cervix
SIR: Systemic inflammatory response
SIRI: Systemic inflammatory index
TC: Tumor/lesion center
TIICs: Tumor-infiltrating immune cells
TSA: Tyramine signal amplification
Tregs: Regulatory T cells
tTIICs: TIICs in the tumor/lesion center
sTIICs: TIICs in the tumor stroma
TS: tumor stroma
