# Supplementary material for: Efficacy of FOXP3+Treg cells combined with platelet in predicting recurrence of cervical cancer: a retrospective study
Source: BMC Womens Health. 2026 Feb 9;26:161. doi: 10.1186/s12905-026-04274-9 (PMC12983664; doi:10.1186/s12905-026-04274-9)
Supplement: Supplementary file 1 — Supplementary Material 1. A flow diagram to summarize the screening process of the study subjects. [file 12905_2026_4274_MOESM1_ESM.pptx]

## Slide 1
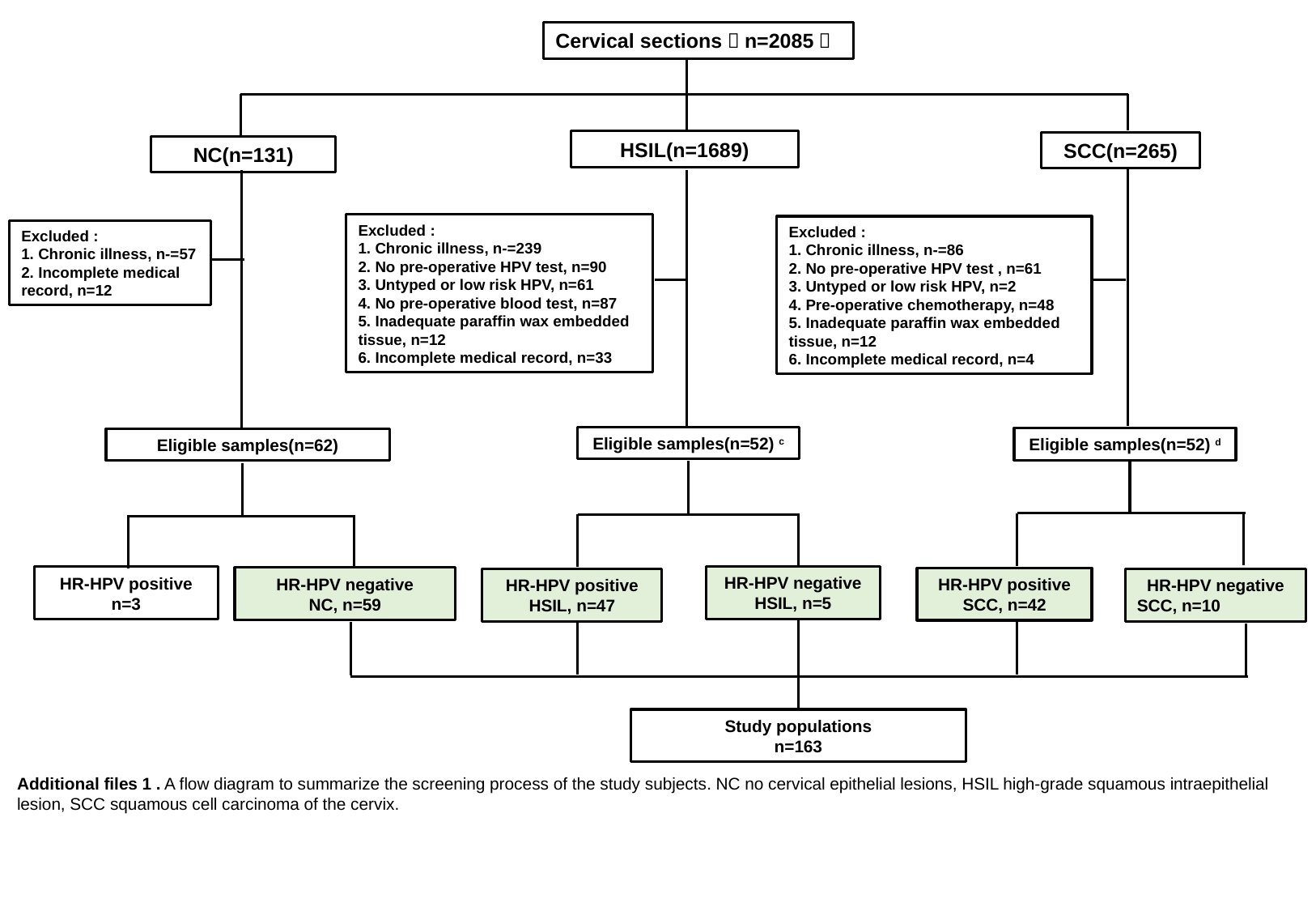

Cervical sections（n=2085）
HSIL(n=1689)
SCC(n=265)
NC(n=131)
Excluded :
1. Chronic illness, n-=86
2. No pre-operative HPV test , n=61
3. Untyped or low risk HPV, n=2
4. Pre-operative chemotherapy, n=48
5. Inadequate paraffin wax embedded tissue, n=12
6. Incomplete medical record, n=4
Excluded :
1. Chronic illness, n-=239
2. No pre-operative HPV test, n=90
3. Untyped or low risk HPV, n=61
4. No pre-operative blood test, n=87
5. Inadequate paraffin wax embedded tissue, n=12
6. Incomplete medical record, n=33
Excluded :
1. Chronic illness, n-=57
2. Incomplete medical record, n=12
Eligible samples(n=52) c
Eligible samples(n=52) d
Eligible samples(n=62)
HR-HPV negative
HSIL, n=5
HR-HPV positive
n=3
HR-HPV negative
NC, n=59
HR-HPV positive
SCC, n=42
HR-HPV negative
SCC, n=10
HR-HPV positive
HSIL, n=47
Study populations
n=163
Additional files 1 . A flow diagram to summarize the screening process of the study subjects. NC no cervical epithelial lesions, HSIL high-grade squamous intraepithelial lesion, SCC squamous cell carcinoma of the cervix.
