# Supplementary material for: Efficacy of FOXP3+Treg cells combined with platelet in predicting recurrence of cervical cancer: a retrospective study
Source: BMC Womens Health. 2026 Feb 9;26:161. doi: 10.1186/s12905-026-04274-9 (PMC12983664; doi:10.1186/s12905-026-04274-9)
Supplement: Supplementary file 2 — Supplementary Material 2. Clinical characteristics of study subjects, n=163. [file 12905_2026_4274_MOESM2_ESM.docx]

**Additional files 2** Clinical characteristics of study subjects, n=163.

|  | no cervical epithelial lesions (NC)  n=59 | | High-grade squamous intraepithelial lesion (HSIL) n=52 | | Squamous cell carcinoma  of cervix (SCC)  n=52 | |
| --- | --- | --- | --- | --- | --- | --- |
| Median age (range), years (*P*=0.236) | | 48 (37-76) | | 45 (27-64) | | 47 (31-76) |
| HR-HPV + | | 0 | | 47 (90.38%) **^c^** | | 42 (80.77%) **^c^** |
| FIGO stage | |  | |  | | < IB2: 23 (44.23%)  ≥ IB2: 29 (55.77%) |
| Tumour differentiation | |  | |  | | Moderate or well: 48 (92.31%)  Poorly: 4 (7.69) |
| Infiltration depth^a^ | |  | |  | | ≤ 1/2: 23 (44.23%)  > 1/2: 29 (55.77%) |
| Lymph node metastasis^b^ | |  | |  | | 8 (15.38%) |
| Perineural infiltration^b^ | |  | |  | | 2 (3.85%) |
| Lymphvascular invasion^b^ | |  | |  | | 11 (21.15%) |

HR-HPV, high-risk types of human papillomavirus; FIGO, International Federation of Gynaecology and Obstetrics. ^a^: half of the cervix wall thickness or less vs over half. ^b^: negative vs positive. ^c^: the positive rate of HPV16 was 65.38% in both HSIL group and SCC group, the positive rate of HPV18 was 0.00% in HSIL group and 7.69% in SCC group, the positive rate of other HPV high-risk subtypes was 25.00% in HSIL group and 7.69% in SCC group.
