# Supplementary material for: Efficacy of FOXP3+Treg cells combined with platelet in predicting recurrence of cervical cancer: a retrospective study
Source: BMC Womens Health. 2026 Feb 9;26:161. doi: 10.1186/s12905-026-04274-9 (PMC12983664; doi:10.1186/s12905-026-04274-9)
Supplement: Supplementary file 3 — Supplementary Material 3. Antibodies used for immunohistochemistry (IHC) and multiplex immunofluorescence (mIF). [file 12905_2026_4274_MOESM3_ESM.docx]

**Additional files 3.** Antibodies used for immunohistochemistry (IHC) and multiplex

immunofluorescence (mIF).

| Antibody | Target | Source | Clonality | Catalog # | Vendor | Dilution |
| --- | --- | --- | --- | --- | --- | --- |
| Anti-CD3 | Pan T cells | Rabbit | monoclonal | ab16669 | Abcam | 1:100 |
| Anti-CD4 | CD4^+^ T helper cells | Rabbit | monoclonal | Ab133616 | Abcam | 1:500 |
| Anti-CD8 | CD8^+^ cytotoxic T cells | Mouse | monoclonal | 66868-1-Ig | Proteintech | 1:1000 |
| Anti-CD20 | B cells | Rabbit | monoclonal | ab78237 | Abcam | 1:200 |
| Anti-CD68 | Pan Macrophages | Mouse | monoclonal | ab955 | Abcam | 1:1500 |
| Anti-CD163 | M2 macrophages | Rabbit | monoclonal | ab182422 | Abcam | 1:400 |
| Anti-CD11b | MDSCs | Rabbit | monoclonal | ab133357 | Abcam | 1:2000 |
| Anti-FOXP3 | Regulatory T cells (Treg) | Mouse | monoclonal | ab20034 | Abcam | 1:300 |
| Anti-PD-L1 | PD-L1 | Rabbit | monoclonal | ab213524 | Abcam | 1:100 |
| Anti-PD1 | PD1 | Mouse | monoclonal | ab52587 | Abcam | 1:50 |
| Anti-NFAT5 | NFAT5 | Mouse | monoclonal | sc-398171 | Santa Cruz | 1:50 |
