# Supplementary material for: Efficacy of FOXP3+Treg cells combined with platelet in predicting recurrence of cervical cancer: a retrospective study
Source: BMC Womens Health. 2026 Feb 9;26:161. doi: 10.1186/s12905-026-04274-9 (PMC12983664; doi:10.1186/s12905-026-04274-9)
Supplement: Supplementary file 4 — Supplementary Material 4. Representative images to demonstrate the delineation of the TC and TS areas in HSIL (a) and SCC (b) tissue samples. [file 12905_2026_4274_MOESM4_ESM.pptx]

## Slide 1
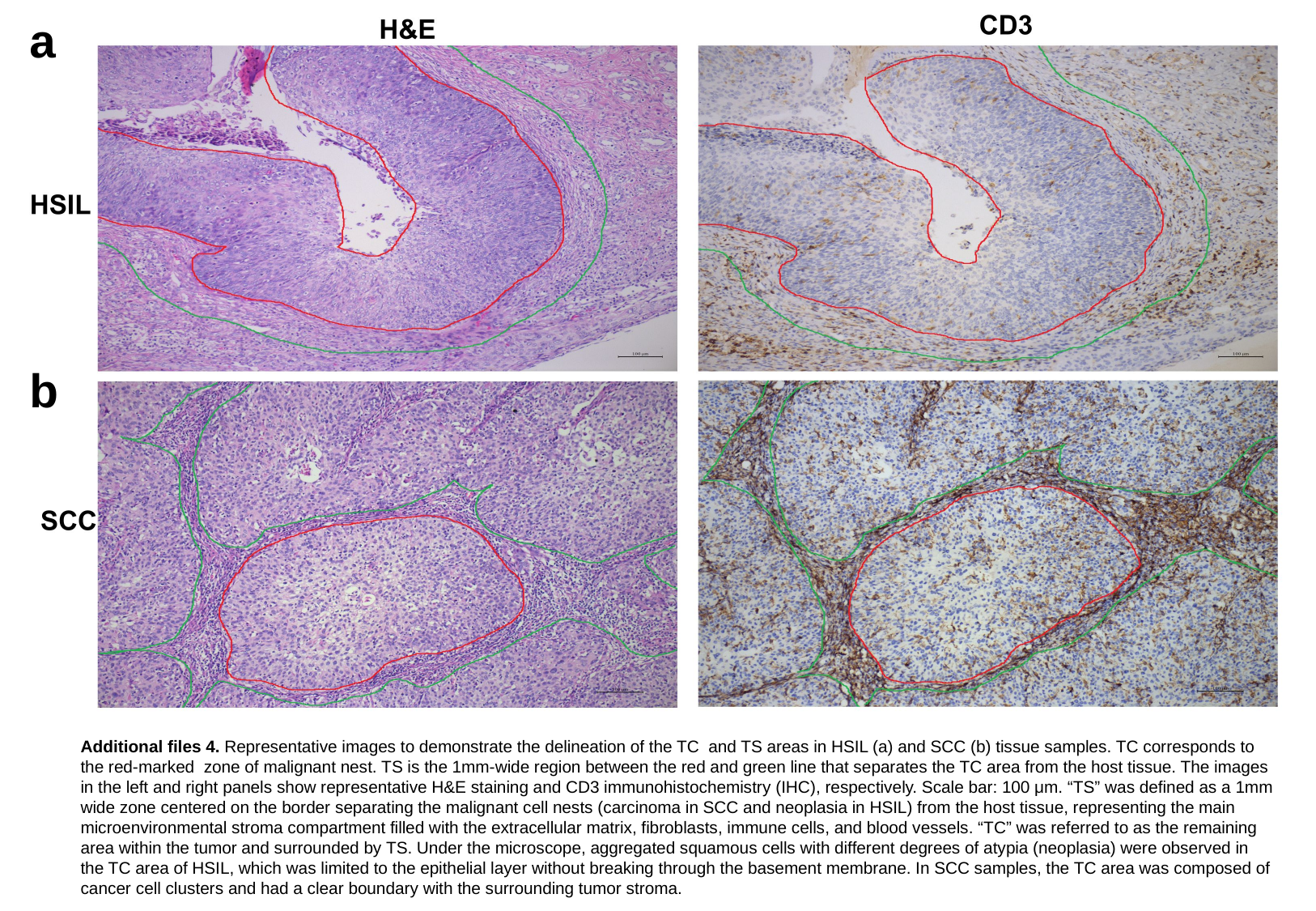

a
b
Additional files 4. Representative images to demonstrate the delineation of the TC and TS areas in HSIL (a) and SCC (b) tissue samples. TC corresponds to the red-marked zone of malignant nest. TS is the 1mm-wide region between the red and green line that separates the TC area from the host tissue. The images in the left and right panels show representative H&E staining and CD3 immunohistochemistry (IHC), respectively. Scale bar: 100 μm. “TS” was defined as a 1mm wide zone centered on the border separating the malignant cell nests (carcinoma in SCC and neoplasia in HSIL) from the host tissue, representing the main microenvironmental stroma compartment filled with the extracellular matrix, fibroblasts, immune cells, and blood vessels. “TC” was referred to as the remaining area within the tumor and surrounded by TS. Under the microscope, aggregated squamous cells with different degrees of atypia (neoplasia) were observed in the TC area of HSIL, which was limited to the epithelial layer without breaking through the basement membrane. In SCC samples, the TC area was composed of cancer cell clusters and had a clear boundary with the surrounding tumor stroma.
