# Supplementary material for: Efficacy of FOXP3+Treg cells combined with platelet in predicting recurrence of cervical cancer: a retrospective study
Source: BMC Womens Health. 2026 Feb 9;26:161. doi: 10.1186/s12905-026-04274-9 (PMC12983664; doi:10.1186/s12905-026-04274-9)
Supplement: Supplementary file 5 — Supplementary Material 5. Univariate Kaplan-Meier analysis of Clinicopathologic features, SIR and TIICs (Overall) in SCC patients. [file 12905_2026_4274_MOESM5_ESM.docx]

**Additional files 5**. Univariate Kaplan-Meier analysis of Clinicopathologic features, SIR and TIICs (Overall) in SCC patients.

|  | Variable(cutoff) | Univariate Kaplan-Meier analysis | | | |
| --- | --- | --- | --- | --- | --- |
|  |  | **HR** | **95% CI** | | ***P*** |
| Clinicopathologic features | Age | 0.682 | 0.208 | 2.236 | 0.536 |
|  | HR-HPV | 0.258 | 0.069 | 0.965 | 0.144 |
|  | FIGO stage | 0.326 | 0.010 | 1.066 | 0.066 |
|  | Tumour differentiation | 0.695 | 0.063 | 7.641 | 0.723 |
|  | Infiltration depth | 0.299 | 0.091 | 0.984 | **0.047^*^** |
|  | Lymph node metastasis | 0.439 | 0.080 | 2.393 | 0.199 |
|  | Perineural infiltration | 0.237 | 0.005 | 12.090 | 0.127 |
|  | Lymphvascular invasion | 0.343 | 0.069 | 1.692 | 0.064 |
| SIR | ANC (2798) | 2.421 | 0.625 | 9.378 | 0.122 |
|  | ALC (1953) | 0.181 | 0.055 | 0.592 | **0.013^*^** |
|  | AMC (430.1) | 0.270 | 0.076 | 0.957 | **0.020^*^** |
|  | PLT (242.5) | 5.047 | 1.373 | 18.540 | **0.015^*^** |
|  | NLR (2.377) | 5.845 | 1.717 | 19.890 | 0.053 |
|  | LMR (4.005) | 0.522 | 0.144 | 1.898 | 0.384 |
|  | PLR (112.6) | 15.340 | 4.579 | 51.410 | **0.000^*^** |
| TIICs (Overall) | CD3 (47.25) | 0.000 | -1.000 | -1.000 | 0.082 |
|  | CD4 (81.00) | 0.211 | 0.063 | 0.722 | **0.008^*^** |
|  | CD8 (81.38) | 2.262 | 0.647 | 7.916 | 0.277 |
|  | CD20 (60.75) | 0.370 | 0.103 | 1.332 | 0.050 |
|  | CD68 (33.75) | 13.530 | 0.125 | 1468.000 | **0.000^*^** |
|  | CD163 (83.25) | 0.408 | 0.071 | 2.329 | 0.143 |
|  | CD11b (35.63) | 0.608 | 0.160 | 2.310 | 0.505 |
|  | FOXP3 (31.25) | 0.245 | 0.073 | 0.820 | **0.023^*^** |

* *P*<0.05. SIR, systemic inflammatory response; TIICs, tumour-infiltrating immune cells; SCC, squamous cell carcinoma of cervix; HR-HPV, high-risk types of human papillomavirus; FIGO, International Federation of Gynecology and Obstetrics; ANC, absolute neutrophil count; ALC, absolute lymphocyte count; AMC, absolute monocyte count; PLT, absolute platelet count NLR, neutrophil-to-monocyte ratio; LMR, lymphocyte-to-monocyte ratio; PLR, platelet-to-lymphocyte ratio; HR, Hazard Ratio (Logrank); 95% CI, 95% confidence interval.
